# Measuring Public Preferences for Changes in the Health Insurance Benefit Package Policies in Iran: A Survey Approach

**Published:** 2020-05

**Authors:** Efat MOHAMADI, Alireza OLYAEEMANESH, Arash RASHIDIAN, Abbas RAHIMI FOROUSHANI, Ali HASSANZADEH, Mojtaba HASUMI, Mohammadreza MOBINIZADEH, Sara MOHAMADI

**Affiliations:** 1. Health Equity Research Center (HERC), Tehran University of Medical Sciences, Tehran, Iran; 2. National Institute for Health Research, Tehran University of Medical Sciences, Tehran, Iran; 3. Department of Health Management and Economics, School of Public Health, Tehran University of Medical Sciences, Tehran, Iran; 4. Department of Epidemiology and Biostatistics, School of Public Health, Tehran University of Medical Sciences, Tehran, Iran; 5. Health Insurance Organization of Iran, Tehran, Iran; 6. Department of Health Economics, School of Health Management and Information Sciences, Iran University of Medical Sciences, Tehran, Iran

**Keywords:** Public preferences, Benefit package, Insurance, Survey, Iran

## Abstract

**Background:**

This study aimed to identify the public preference in health services, the principles that Iranian people consider important, and the aspects of tradeoffs between different values in resource allocation practices.

**Methods:**

This quantitative study was conducted to investigate public preferences on Health Insurance Benefit Package (HIBP) in 2017. A structured questionnaire was used for data collection, including the preferences of the people who live in Tehran, were above 18 year, and were covered by basic insurance for the HIBP contents and premium. The sample size was calculated 430 subjects and SPSS Statistics was used for data analyzing.

**Results:**

81.6% of the sample population agreed with government allocating more money to the health sector compared to other sectors and organizations and 55% were willing to pay higher premiums for expanding the HIBP coverage. The highest and lowest score regarding prioritization of budget allocation between health services was related to hospitalization services (28.6%) and rehabilitation services (1.6%), respectively. The first priority of respondents regarding health care and life cycle, was “prevention in newborns” (15.9%), the second priority was “prevention in children” (14.6%), the third priority was “prevention in adults” (9.5%), and the last priority was “short-term care in newborns” (0.9%).

**Conclusion:**

Iranian people believe that not only the principle of health maximization but also equal opportunities to access health care and a fair allocation of resources should be considered by authorities for effective health insurance policymaking. In this case, given the scarcity of resources, setting priorities for alternative resources is inevitable.

## Introduction

Increased population ageing and improvements in curative procedures due to advances in medical technology have led to increasing demand for healthcare services worldwide (1), resulting in a growth in healthcare expenditures while the budget is more and more limited. Considering these budget constraints, policies have to be made for sustainable healthcare financing (2). One of the crucial issues in this regards is the design of insurance benefit package; so far, developing countries, have been facing with several challenges in this field (3–5). Iran’s Ministry of Health and Medical Education has planned to prioritize health services in a robust framework in recent years.

In some countries, such as New Zealand and Sweden, to enhance the transparency and legitimacy of the decision making, the criteria for funding decisions are available for public (6). Moreover, an increasing number of countries, including the UK and Ireland, have started to involve the general public (7).

Patient preferences are increasingly considered important in decision-making related to healthcare (8). Generally, clinicians’ ideas for using guidelines and technologies are determined by some factors, like personal beliefs (9). However, patient-related factors are considered as important factor in this regard. Acknowledging the importance of the patients’ ideas, increasing evidence in this regard has become available in recent years (9).

While the value of using patient preferences has been recognized so far, but some challenges have been identified in this regard, for example, many patients have limited experience in thinking about the preferences (10). These problems derive from various issues and it will be vague on how patient preferences values should be traded-off with other factors (11).

Considering the evidence related to patient preferences for decisions making is important due to some following reasons; the most of the criteria for evaluating health system do not consider patients’ perspectives; It can be used as the good source of information; the research on patient preferences can influence medical decisions and it is ethical to consider patients’ views (11).

In Iran, few studies have explored public preferences regarding health care. Hence, this study aimed to identify the principles that Iranian people consider important in health insurance benefit package (HIBP).

## Methods

This qualitative study was conducted to investigate public preferences for the HIBP in 2017. The preferences of the public about different combinations of the HIBP were assessed. A structured questionnaire was used to collect preferences of the people who lived in Tehran, were above 18 year, and were covered by basic health insurance at the time of the study.

### Designing and Developing the Questionnaire

At first, we needed to clarify aspects of the HIBP, so Focus Group Discussions (FGD) was established to define the HIBP attributes. Participants in this FGD were key informants on health insurance and health financing fields. In the next step, we reviewed the studies that conducted on the same subject to retrieve those methods in data collection and data analysis and also structure of questionnaires. Then, an initial draft of the questionnaire was formulated base on the outcomes of FGD and literature. By the end, demographic questions were added and the final draft of the questionnaire was prepared. These questionnaire consists of four parts: 1. Socio-demographic information, 2. HIBP policies, 3. Budget allocation among health service groups, 4. Health care and life cycle matrix priorities.

### Validity of the Questionnaire

To assess the content validity of the questionnaire, individual questions were reviewed by seven experts in the field of health insurance. First, the Content Validity Index (CVI) was calculated for each item separately, indicating a CVI range of 0.79 to 1. Then, the Content Validity Ratio (CVR) was calculated for each question. According to the Lawshe’s method for assessing content validity, in case of any doubts, an item perceived to be “essential” by more than half of the panelists has some degrees of content validity (12). Therefore, since all the questions were considered “essential” by more than half of the people, none of them was eliminated. Finally, after matching the content validity index with content validity ratio and clarifying the questions, they were included in the final draft of the questionnaire.

### Reliability of the Questionnaire

For this purpose, the questionnaires were given to 20 subjects. The Cronbach’s alpha was used to assess the reliability of the questionnaire; its value was 0.806, indicating the acceptable reliability of the questionnaire.

On the other hand, assessment of each question showed that their elimination would not result in a significant increase or decrease in the reliability coefficient. Thus, all the questions had good internal consistency both in the indicator and in the general level.

### Sample size calculation

Since the target population was 7,148,794 people, the Cochran formula with a 5% sampling error was applied to determine the sample size. By using this method, the sample size was calculated as 430 subjects. In this study, assuming a maximum variance in the sample at a confidence level of 95% ($p=q=\frac{1}{2}$ , *S*^2^ = *pq* = 0.25) and acceptable error of 5%, the sample size was calculated as follows:

$$n=\frac{Nt^{2}s^{2}}{Nd^{2}+t^{2}s^{2}}\approx 430$$

Where:

t_0.975_ = 1.96 ≈ 2

The following sentences describe why we took S^2^ = 0.25. Our study was multipurpose, indicating that we had to assess more than two or three variables to meet the objectives. Thus, we had to optimize the sample size based on every single variable. A variable that has more dispersion between measurements requires a larger sample size than other variables. So, if the sample size is optimized for that variable, it is optimized for other variables, as well.

### Sampling method

For sampling, we used a combination of proportional to size stratified sampling and systematic randomized sampling methods. Based on the proportional to size stratified sampling method, the sample size of each region (22 regions) was determined concerning the total population of adults aged 18 year and over, living in Tehran (Table 1). Then, in each region, a number of blocks were determined and the sample blocks were selected. In the next step, using the systematic randomized sampling method and based on the number of samples in each region and blocks, individuals aged 18 year and over in selected blocks were interviewed face to face, and the questionnaires were completed.

**Table 1:** Number of people aged 18 yr and over and sample size in each region

***Regions***	***Regional population***	***Sample population***	***Regions***	***Region’s population***	***Sample population***
NO1	379,962	20	NO12	248,048	13
NO2	608,814	33	NO13	245,724	13
NO3	290,726	15	NO14	433,432	26
NO4	822,580	44	NO15	644,259	34
NO5	344,019	18	NO16	291,169	16
NO6	237,292	13	NO17	256,022	14
NO7	310,184	16	NO18	317,188	17
NO8	378,725	20	NO19	249,786	14
NO9	165,903	9	NO20	335,634	18
NO10	315,619	17	NO21	159,793	8
NO11	275,241	15	Region NO22	108,674	7
Total Population			7,148,794		
Sample Population			430		

### Data analysis

After collecting the questionnaires, the data were categorized and analyzed by SPSS (Chicago, IL, USA) software 20. The descriptive statistics was carried out for each part of questionnaire and central tendency were measured.

### Ethical Approval

The confidentiality of questionnaires information had been assured. Also this study received the ethical code from Tehran University of Medical science: IR.TUMS.REC.1395.2517.

## Results

### Analysis of demographic variables

430 people (100% of the participants) completed the questionnaires. The mean age of the participants was 45 yr (range: 18–90 yr) (Table 2).

**Table 2:** Frequency of demographic variables of respondents

***Variable***	***Group***	***Frequency***	***Percentage***
Gender	Male	215	50
	Female	215	50
Age(yr)	18 – 24	61	14.2
	25 – 29	55	12.8
	30 – 39	76	17.7
	40 – 49	53	12.3
	50 – 59	75	17.4
	60 – 69	68	15.8
	≥ 70	42	9.8
Level of education	Illiterate	32	7.4
	Primary School	56	13
	Secondary School	46	10.7
	High School	11	2.6
	Diploma	133	30.9
	BS and higher level	152	35.3
Monthly costs (Rials)	Less than 500,000	7	1.6
	More than 500,000 – 1 million	65	15.1
	More than 1 million – 1.5 million	92	21.4
	More than 1.5 million – 2 million	111	25.8
	More than 2 million – 3 million	79	18.4
	More than 3 million – 4 million	24	5.6
	> 4 million	10	2.3
	No reply	42	9.8

### Analysis of health-related variables

In this step, the participants were categorized based on their health insurance organization, the assessment of their health status (very good, good, fairly good, fairly poor, poor, very poor, and I don’t know), a family member suffering from chronic diseases such as diabetes, hypertension, heart disease, cancer, respiratory diseases, etc. (yes, no, I don’t know), hospitalization of a family member in the past 6 months (yes, no, I don’t know), and a family member visiting the physician in the past 6 months (yes, no, I don’t know) (Table 3).

**Table 3:** Frequency of health-related variables

***Variable***	***Group***	***Frequency***	***Percentage***
Type of health insurance	Social Security Insurance	292	67.9
	Health Insurance Organization	80	18.6
	Military Health Insurance	28	6.5
	Imam Khomeini Relief Committee	2	0.5
	Others	28	6.5
Own health status assessment	Very good	49	11.4
	Good	179	41.6
	Fairly good	137	31.9
	Fairly poor	41	9.5
	Poor	18	4.2
	Vary poor	5	1.2
	I don’t know	1	0.2
A family member suffering from chronic diseases	Yes	239	55.6
	No	191	44.4
	I don’t know	332	77.2
Hospitalization of a family member in the past 6 months	Yes	97	22.6
	No	0	77.2
	I don’t know	1	0.2
A family visiting a physician in the past 6 months	Yes	345	80.2
	No	84	19.6
	I don’t know	1	0.2

About 68% of the sample population was covered by Social Security Insurance and 18.6% was covered by Health Insurance Organization. The percentage of participants who assessed their health condition as “good” was the highest among all conditions with 41.6%. Given that 57% of the sample population was under the age of 50 (18–50 yr), this level of health status assessment was well expected. However, 1.2% of the people reported their health condition as “very poor”. A significant percentage of the study population (9.8%) was above the age of 70, so this percentage of “very poor” assessment seems reasonable.

### Participants’ opinions about basic health insurance policies

In this section, the participants were asked to express their comments about basic insurance policies on a Likert scale from “totally agree” to “totally disagree” (Table 4).

**Table 4:** Participants’ comments about basic insurance policies

***Questions***		***Totally agree***	***Agree***	***Disagree***	***Totally disagree***	***No idea***	***Total***
The government should provide more money to the health sector than the other governmental agencies and institutions.	frequency	124	227	24	3	52	430
	percent	28.8	52.8	5.6	0.7	12.1	100
I am willing to pay more when I am healthy and pay less in time of using health services.	frequency	65	193	109	9	54	430
	percent	15.1	44.9	25.3	2.1	12.6	100
I am willing to pay more for increasing insurance coverage (health and medical services coverage)	frequency	49	191	121	17	52	430
	percent	11.4	44.4	28.1	4.0	12.1	100
I believe that the needs and demands of people for health care services covered by insurance companies are taken into account.	frequency	31	123	168	48	60	430
	percent	7.2	28.6	39.1	11.2	14.0	100

According to our results, 81.6% of the sample population agreed with the government allocating more budgets to the health sector compared to other sectors and organizations, 59% were willing to pay higher premiums to pay less at the time of receiving health services, and 55% were willing to pay higher premiums for expanding the coverage of the health services package.

### Budget allocation by respondents among health service groups

**Fig. 1:**
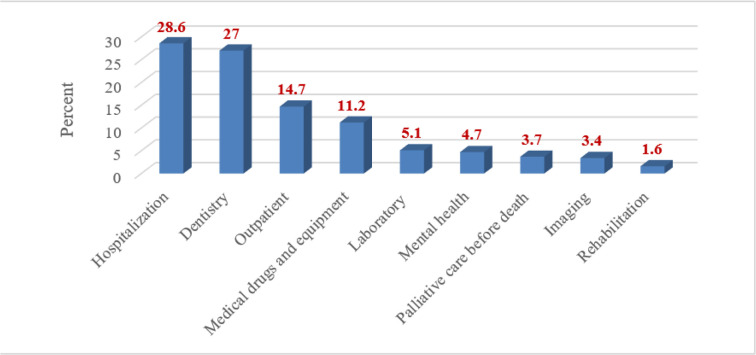
Budget allocation among health services groups

In this section, the participants, assuming that they had a certain budget, were asked to allocate all of it to designated health service groups, including hospitalization services, dentistry services, outpatient services, medical drugs and equipment, laboratory services, mental health related services, palliative care services before death, imaging services (MRI, CT scan, radiography, etc.), and rehabilitation services (physiotherapy, occupational therapy, etc.). Figure 1 presents the average score of participants regarding prioritization of budget allocation among health services groups.

The results of prioritizing health services for budget allocation showed that hospitalization and dental services had the highest priority, receiving 28.6% and 27% of the total vote, respectively. In this case, rehabilitation services (1.6%) had the lowest priority.

### Participants’ priorities based on health care and life cycle matrix

The purpose of this section was to evaluate the people’s preferences regarding the allocation of health services according to different age groups using a matrix. In this matrix, one axis corresponded to the human life cycle, including infancy, childhood, adulthood, and old age. The other axis represented the type of health care, including vital care (special care, organ transplantation, open and severe surgery, burns and emergency services), short-term care (visits and short hospitalizations), long-term care (elderly and palliative care), and preventive care (vaccination, check-up, and screening). The participants were asked to prioritize matrix houses from the top priority [1] to the last priority [16]. Based on the average score given to each health care service in different life cycles, the services were prioritized from high priority to low priority (Table 5). The numbers in parentheses reflect the percentage of each item selected as the priority by participants.

**Table 5:** Participants’ priorities based on health care and life cycle matrix

***Variable***	***Life cycle***				

Type of Health services	infancy	childhood	adulthood	old age
vital care	Average Priority (5.7)	Average Priority (5.3)	Average Priority (8)	Average Priority (8.1)
Long-term care	Average Priority (2.5)	Low Priority (2.5)	Average Priority (4.2)	Average Priority (6.9)
Short-term care	Low Priority (0.9)	Average Priority (2.6)	Average Priority (3.3)	Low Priority (2.4)
Preventive care	High Priority (15.9)	High Priority (14.6)	High Priority (9.5)	Average Priority (7.6)

The results of the healthcare and life cycle matrix showed that based on the participants’ opinions, the first three priorities were preventive care services in infants (15.9%), children (14.6%), and adults (9.5%), while short-term care for infants and elderlies (0.9%) and long-term care for children (2.5%) had the lowest priority.

## Discussion

In this study, about 68% of the sample population was covered by Social Security Insurance and 18.6% was covered by Health Insurance Organization; while the average population of Iran (53%) is covered by Health Insurance Organization (13). Most of the population covered by this insurance is villagers and nomads while this study population comprised people in Tehran (with no villagers and nomads), the composition of our sample does seems logical.

The percentage of participants who assessed their health condition as “good” was the highest among all conditions with 41.6%. Given that 57% of the sample population was under the age of 50 (18–50 yr), this level of health status assessment was well expected. However, 1.2% of the people reported their health condition as “very poor”. The results showed that a significant percentage of the study population (9.8%) was above the age of 70, so this percentage of “very poor” assessment seems reasonable.

Of all participants in the study, 44.4% indicated that at least one of their family members suffered from chronic diseases. In a study, 20.7% of the participants reported that they suffered from a chronic disease (14). The difference between our study and the study by Larijani et al (14) may be related to the study population. In our study, the questions were related to the whole family of the participants while they asked questions about the participants themselves. It also worth noting that 43% of the global burden of diseases is associated with major non-communicable and chronic diseases (14).

The results of hospitalization frequency indicated that 22.6% of the people had at least one family member hospitalized least once in the past 6 months. About 80.2% of people also stated that one of their family members visited a physician at least once in the past 6 months. According to the national health accounts statistics (15), the proportions found in this study appear to be reasonable.

The sample population mostly agreed with the government allocating more budgets to the health sector compared to other sectors and organizations. This could be possible either by increasing the total budget share of the health sector or the premium paid by the public.

Half of the subjects believed that the people’s demands for health services covered by insurers were not taken into consideration. This rate was expected to be lower, considering that more than 80% of the services provided in the health system are covered by basic insurances. This finding could be due to non-coverage of some medical technologies (medications and interventions) for which no certain tariffs have even been set, or due to the low coverage of basic insurances in private hospitals; in both cases, the patients have to pay the total charge of received services. The most pessimistic reason for this belief is the low level of participants’ knowledge about the subject asked. However, in general, indeed, the public view is not directly considered in service coverage.

For budget allocation, hospitalization and dental services had the highest priority and rehabilitation services had the lowest priority. The high cost of dental services and lack of insurance coverage made these services a high priority. Moreover, the low priority of rehabilitation services can be due to that fewer people usually utilize these services compared to other services. However, considering the high risks associated with accidents and also the need for rehabilitation services in certain patients, which would lead to catastrophic expenditures, it is suggested that effective policies should be made and implemented to cover these services and deliver them to the patients appropriately. Although dental services are less frequently used than services like laboratory services and medicines, people need this type of services to be covered by insurance companies due to their high costs. These services should be defined in the first-level service package, provided that they have an acceptable quality to persuade people to utilize them, to prevent high costs of dental treatments.

In other related literature, prevention mostly was preferred to cure for disease in young adults, and severe diseases in total population too (16). Moreover, lifesaving interventions are more important than those which are life-extending or enhancing QALY (17).

The healthcare and life cycle matrix showed that the first three priorities were preventive care services in infants, children, and adults, while short-term care for infants and elderlies and long-term care for children had the lowest priority. People believed that preventive care services are more important than vital, short-term, and long-term care services. We expected that vital care for adults be prioritized as one of the first three priorities. However, social marketing in that media about health care services, which places more emphasis on prevention than treatment, is one of the most important factors behind this finding. The results of the present study are similar to a report which preventive care services for infants, children, and adults of Oregon were selected as the first three priorities and long-term care services for infants and children had the lowest priority (18). Children care related services and special pain and palliative care services before death were the first and second priority respectively, and services related to infertility and elderlies above 75 year had the lowest priorities (19).

## Conclusion

On the base of this research, equal opportunities to access health care and a fair allocation of resources should be considered by health authorities. Moreover, there is a capacity to define an “essential services package” financed through the government, and to define a “higher-level service package” financed by people. These packages should be defined based on people’s preferences with different premiums to create different choices for them.

The limitation of this study was related to sample size. We could not add people and spread sample throughout the country because of time and cost constraints. In this regard, the research group has tried to sample all areas of Tehran so that it is available from all sections of the sample society with different socio-economic level of people.

## Ethical considerations

Ethical issues (Including plagiarism, informed consent, misconduct, data fabrication and/or falsification, double publication and/or submission, redundancy, etc.) have been completely observed by the authors. The confidentiality of Topic Guide information has been assured.
